# A small-cohort study on tumor recurrence and surgery-related complications associated with proximal fibular tumors and the potential utility of biopsy

**Published:** 2021-02-25

**Authors:** Tao Sun, Michal Heger, Lingxiang Wang, Mengjing Niu, Shuman Han, Xiaoran Zhang, Haitao Zhao, Wenjuan Wu

**Affiliations:** ^1^Department of Orthopedic Surgery, Third Hospital of Hebei Medical University, Shijiazhuang, Hebei Province 050051, PR China; ^2^Department of Pharmaceutics, Jiaxing Key Laboratory for Photonanomedicine and Experimental Therapeutics, College of Medicine, Jiaxing University, Jiaxing, Zhejiang, PR China; ^3^Laboratory Experimental Oncology, Department of Pathology, Erasmus MC, Rotterdam, Netherlands; ^4^Department of Gynecology, Fourth Hospital of Hebei Medical University, Shijiazhuang, Hebei Province 050011, PR China; ^5^Department of Radiology, Third Hospital of Hebei Medical University, Shijiazhuang, Hebei Province 050051, PR China

**Keywords:** biopsy, bone cancer, China, epidemiology, histology and pathology, safety

## Abstract

**Aim:**

The aim of the study was to assess the incidence of tumor recurrence, iatrogenic peroneal nerve injury, and wound healing problems in a small cohort of patients with proximal fibular tumors who had undergone surgery, and to determine the relative risk of pre-operative biopsies on these outcome variables.

**Methods:**

The study entailed a retrospective single-center analysis of patients with a histologically confirmed tumor in the proximal fibula who had undergone surgery at our institute between 2004 and 2019 (*n* = 66). The accuracy of diagnosis based on pre-operative biopsy (*n* = 10) was compared to the histological diagnosis based on resection specimens. The association between pre-operative biopsy and patient demographics and medical history as well as tumor recurrence, iatrogenic peroneal nerve injury, and impaired wound healing was analyzed statistically. The data were presented against a backdrop of bone cancer incidence and 5-year survival rates in China.

**Results:**

Recurrence, iatrogenic peroneal nerve, and wound healing issues were identified in 5 (7.6%), 8 (12.1%), and 6 patients (11.0%), respectively. A biopsy was acquired from ten of 66 patients. The pre-operative biopsy diagnostic accuracy rate was 100%. Males had an 11.2-fold higher risk of undergoing pre-operative biopsy than females (95% CI, 1.3-94.1; *P* = 0.013). Pre-operative biopsies were 11.2 times more likely to be obtained from patients with malignant and benign aggressive tumors in the proximal fibula compared to benign tumors (95% CI, 1.1-63.1; *P* = 0.013). Patients who had undergone biopsy were 12.4 times more likely to receive Type I or Type II *en bloc* resection (95% CI, 1.5-104.3; *P* = 0.006) and had a 7.6-fold greater chance to have impaired wound healing (95% CI, 1.3-45.1; *P* = 0.040), which was observed mainly in osteosarcoma patients. There were no significant associations of biopsy with tumor recurrence (*P* = 0.162) and iatrogenic peroneal nerve injury (*P* = 0.095).

**Conclusions:**

Biopsy of proximal fibular lesions does not increase the risk for tumor recurrence and iatrogenic peroneal nerve injury but may be associated with post-surgical wound healing problems. This is particularly relevant for male patients and malignant and aggressive benign lesions, where biopsies are considerably more likely to be acquired to guide diagnosis and clinical management. Due to the relatively low incidence of this cancer type and the scarcity of pre-operative biopsies, larger cohort studies are warranted to validate the results.

**Relevance for patients:**

Patients who present with proximal fibular tumors are often young. Depending on the diagnosis of the bone cancer subtype, the surgical intervention may entail highly invasive and risky procedures. Taken together, it is imperative to ensure accurate diagnosis of the bone cancer subtype to prevent unnecessary procedures. Diagnostic accuracy can be increased by acquiring a histological specimen of the malignant bone tissue. However, it is currently not completely established whether bone biopsies in the proximal fibula can be safely performed and whether such biopsies lead to seeding metastases. Because of the rarity of these tumors and procedures, studies are needed even when these entail a small sample size.

## 1. Introduction

Proximal fibular tumors are rare and mostly benign. However, malignant tumors in this anatomical location account for significant morbidity and mortality [1]. Proper clinical management of proximal fibular lesions is predicated on accurate diagnosis. Consequently, the reliability of symptoms at presentation, clinical chemistry, and imaging has been investigated in the context of predicting the benign or malignant nature of the lesion [2]. When aggressive malignant and benign tumors are suspected, especially in case of osteosarcoma, a biopsy is recommended and commonly performed before resection to confirm the diagnosis [3,4].

However, the threshold to biopsy proximal fibula tumors is high. The main concern with biopsies is directly or indirectly increasing the risk of iatrogenic nerve injury due to the anatomical adjacency of the proximal fibula to superficial and deep peroneal nerves [4]. Second, a biopsy may cause local recurrence, infection, and wound healing complications. Finally, the diagnostic accuracy of proximal fibular biopsies is elusive and the added value to steering the type of intervention or surveillance of proximal fibular lesions is also not clear, given the rarity of these tumors, the paucity of studies on this subject, and the lack of putative guidelines.

Proximal fibular tumor cases with confirmatory histological diagnosis were therefore retrospectively reviewed to assess the incidence of peroneal nerve injury and wound healing issues as well as tumor recurrence.

## 2. Materials and methods

### 2.1. Patients, exclusion and inclusion criteria

After institutional review board approval (IRB protocol #2020-022-1), the medical records of 94 patients who had been treated between July 2004 and June 2019 for proximal fibular tumors were retrieved and reviewed. Only cases where the proximal fibular tumors had been histologically confirmed by the pathologist were included in the study. Histopathological confirmation of the malignancy was performed in post-surgical tumor specimens. Histological sections were not re-reviewed for the current study. The medical records, imaging data, and histological analyses were analyzed and processed retrospectively.

Using a standardized data collection form, we retrieved the following information from medical charts: gender, age at diagnosis, laterality involved, presentation of symptoms and signs, biopsy, and surgical management. Any change in symptoms was denoted as changes in the intensity or frequency of the original symptoms or signs and the occurrence of new symptoms or signs. Time from onset to presentation was measured in months. Onset was defined as the time at which the disease or condition was first remarked and at which symptoms first became apparent to the patient. Onset was estimated based on the patient’s personal account during the physician’s anamnesis. Patients were followed through the tumor registry database at our institution. The follow-up routine included examinations of patients every 3 months for the first 2 years after surgery. Thereafter, follow-up was dependent on the patient’s status quo and the pathology results. Patients were not recalled specifically for the study; all data were retrieved from the medical records.

### 2.2. Analysis of bone cancer incidence and survival rates in China

Epidemiological data regarding bone cancer incidence rates and 5-year survival were retrieved from the CI5plus database assembled by the International Agency for Research on Cancer (IARC; World Health Organization) [https://ci5.iarc.fr/CI5plus/Pages/graph4_sel.aspx, accessed 23 October 2020]. The search criteria encompassed bone cancer, both genders, China (5 registries), age-standardized incidence rate, and an age range of 0-85+ years. The IARC database did not contain information on bone cancer survival rates, which were subsequently retrieved from cancer registries in China [5]. Data were processed in GraphPad Prism (GraphPad Software, San Diego, CA, USA).

### 2.3. Statistical analysis

The epidemiological data in regard to incidence of bone cancer in China were tested for normality using a D’Agostino Pearson omnibus test. Next, a paired homoscedastic Student’s t-test was used to assess the difference in incidence between men and women. These analyses were performed in GraphPad Prism.

Descriptive statistics were used to summarize the demographic and clinical data. Data were presented as median with range (minimum and maximum) for continuous variables and as number (N) with percentage for categorical variables. The differences in continuous and categorical variables were assessed using the two-tailed Mann–Whitney U-test or Chi-square test. The association between biopsy and laterality (left, right), symptoms and signs (yes, no), symptom change (yes, no), and histopathological diagnosis (malignant and benign aggressive, benign), intralesional curettage (yes, no), Type I and Type II *en bloc* resection (yes, no), local recurrence (yes, no), and impaired wound healing (yes, no) was analyzed using the Fisher exact test. The relative risk and 95% confidence interval were also calculated. Statistical analyses were performed in SPSS (SPSS, Chicago, IL, USA). *P* ≤ 0.05 was considered statistically significant.

## 3. Results

### 3.1. Patient characteristics

Sixty-six patients with proximal fibular tumors were included in the study. Twenty-eight patients were excluded due to non-surgical treatment or surgery elsewhere. All included patients (*n* = 66) had undergone surgery at our institute. The patient demographics, medical history, and treatment are presented in Table 1.

**Table 1:** Patient demographics, medical history, and treatment-related data (*n*=66)

Age (years)*	18.5 (4-72)
Time from onset to presentation (months)*	33 (0-108)
Gender^†^	
Male	34 (51.5)
Female	32 (48.5)
Laterality^†^	
Right	27 (40.9)
Left	39 (59.1)
Presentation^†^	
Mass	36 (54.5)
Pain	32 (48.5)
Incidental finding on radiographs	9 (13.6)
Peroneal nerve symptoms	8 (12.1)
Palpable pain	21 (31.8)
Elevated skin temperature	7 (10.6)
Symptom change^‡^	13 (19.7)
Histopathological diagnosis^†^	
Benign	42 (63.6)
Benign aggressive	13 (19.7)
Malignant	11 (16.7)
Intervention^†^	
Biopsy	10 (15.2)
Intralesional curettage	6 (9.1)
Marginal resection	27 40.9)
Type I *en bloc* resection	27 (40.9)
Type II *en bloc* resection	6 (6.1)
Recurrence^†^	5 (7.6)
Surgical complications^†^	
Iatrogenic peroneal nerve injury	8 (12.1)

* Values represent the median (range).

† Values represent *n* (%).

‡ Symptom change includes the change in the intensity or frequency of the original symptoms or signs and occurrence of new symptom or signs

### 3.2. Bone cancer epidemiology in China and the study cohort

**Figure 1:**
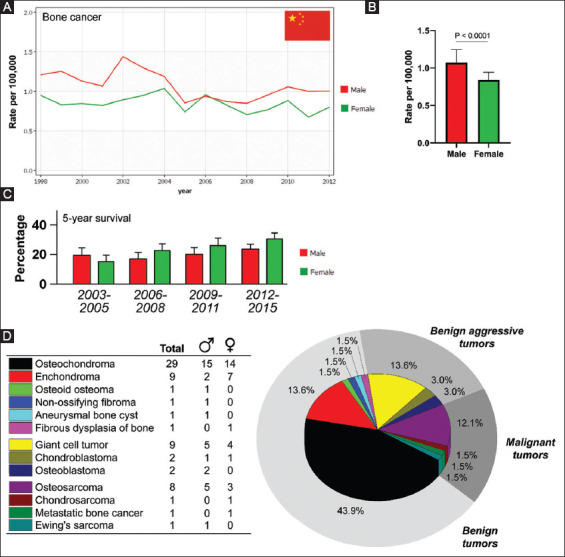
**(A)** Age-standardized incidence of bone cancer in China (1998-2012). Source: World Health Organization | International Agency for Research on Cancer (CI5*plus*), from all available registries in China (Shanghai, Jiashan, Zhongshan, Harbin, Hong Kong). **(B)** Mean ± SD incidence of bone cancer in China (1998-2012) stratified by gender (*n* = 15/group). Analysis based on data from **(A)**. Difference was tested with a Student’s *t*-test. **(C)** Mean ± SD 5-year survival in bone cancer patients stratified per measurement period and per gender. Data obtained from cancer registries in China [5]. **(D)** Overview of the tumor types that make up the proximal fibular lesions in the patient cohort (*n* = 66).

Bone cancer epidemiological data for China were only available for the period 1998-2012 in IARC. The incidence of bone cancer roughly fluctuated around 1/100,000 during the registration period (Figure 1A) and was higher in men than in women (Figure 1B). The incidence was also higher at older age (0-24 y ≈ 25-59 y < 60-85+ y; data not shown). The age-standardized 5-year survival had increased over time (Figure 1C) by 2.0% and 4.9% per year in the period 2003-2015 for men and women, respectively [5], which was attributable to better clinical management of the malignancies [5,6]. Despite improved care, the 5-year mortality associated with bone cancer is the 6^th^ highest for men and women in China [5], underscoring the need for improved diagnostics and monitoring as well as therapeutics and surgical interventions.

An overview of the types of lesions is presented in Figure 1D. Forty-two patients (63.6%) had benign tumors that included osteochondroma, enchondroma, osteoid osteoma, non-ossifying fibroma, fibrous dysplasia of bone, and aneurysmal bone cyst (ABC). Thirteen patients (19.7%) had benign aggressive tumors including giant cell tumor, chondroblastoma, and osteoblastoma. Eleven patients (16.7%) had malignant tumors including osteosarcoma, chondrosarcoma, Ewing’s sarcoma, and metastatic bone cancer. The most common benign tumors in the proximal fibula were osteochondromas (29 cases, 43.9%) and enchondromas (nine cases, 13.6%). The most common benign aggressive tumor was giant cell tumor (nine cases, 13.6%) and the most common malignant tumor was osteosarcoma (eight cases, 12.1%). ABC in the absence of other lesions was diagnosed in one patient (1.5%) and in the presence of other tumors in four patients (6.1%).

The median follow-up was 2.6 years (range: 5 m - 6.5 years). The most common symptoms at presentation were a palpable mass (*n* = 36; 54.5%) and pain (*n* = 32; 48.5%). Neurological symptoms and signs with peroneal nerve compression were observed in eight patients (12.1%) (Table 1). These are in line with previously reported symptoms in a cohort of 112 patients with malignant proximal fibular tumors [7].

### 3.3. Surgical interventions

The intervention was decided by the operating orthopedic oncologist based on the type and severity of the tumor as well as the pre-operative biopsy. The surgical interventions included intralesional curettage, marginal resection, and Type I or Type II *en bloc* resection of the proximal fibula (Table 1). Intralesional curettage was indicated and performed in six patients (9.1%) with intra-osseous benign and benign aggressive lesions. Marginal resection was performed in 27 patients (40.9%) with an extra-osseous benign tumor such as osteochondroma. Type I and Type II *en bloc* resection, as described by Malawer [8], was indicated for those with large benign, benign aggressive, and malignant lesions and performed in 27 (40.9%) and 6 (9.1%) patients, respectively.

### 3.4. Recurrence

Local recurrences were diagnosed in five patients (7.6%) from 2 months to 2 years after the first surgery (Table 1). Osteosarcoma recurred in four patients and a giant cell tumor recurred in one patient. The recurrences led to an above-knee amputation in two patients with osteosarcoma.

### 3.5. Iatrogenic nerve injury

Iatrogenic peroneal nerve injuries following surgery were diagnosed in eight patients (12.1%) and entailed three new cases and five aggravated cases (Table 1). Three patients had permanent peroneal nerve injury because they underwent Type II *en bloc* resection, which entailed peroneal nerve sacrifice to fulfill the resection margin. Five of the eight iatrogenic peroneal nerve injuries (62.5%) resolved or restored to preoperative level. Four of these five patients had been subjected to Type I *en bloc* resection and one of these patients had undergone a marginal resection. Of the three new cases, two patients had received Type I *en bloc* resection and the third patient a marginal resection.

### 3.6. Impaired wound healing

Wound healing issues were observed in seven patients (10.6%) due to incision drainage. Four of seven patients had undergone Type II *en bloc* resection, the other three patients a Type I *en bloc* resection. One of the patients with wound healing problems developed skin necrosis.

### 3.7. Pre-operative biopsies

**Figure 2:**
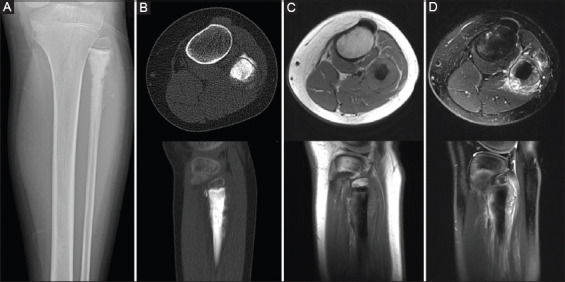
Exemplary radiographic imaging of an osteosarcoma in the left proximal fibula in a 10-y old boy (Table 2, case 9). **(A)** Plain radiograph shows “cloud-like” osteogenic destruction in medullary and cortical bone with Codman triangle and soft tissue mass in the proximal fibula. **(B)** Transverse (top) and sagittal (bottom) CT images add details of osteogenic destruction in the proximal fibula. Transverse CT image shows a lamellate reaction. **(C)** Transverse (top) and sagittal (bottom) T_1_-weighted MRI reveals a low signal intensity mineralized compartment in medullary and cortical bone and an intermediate signal intensity soft-tissue non-mineralized compartment around the proximal fibular tumor. **(D)** Transverse (top) and sagittal (bottom) T_2_-weighted MRI shows a low signal intensity mineralized compartment in medullary and cortical bone, a medium-high signal intensity soft-tissue non-mineralized compartment around the fibular tumor, and high signal intensity peritumoral edema.

**Figure 3:**
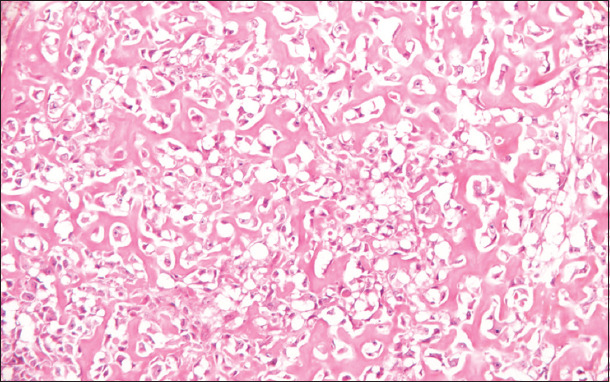
Histological section of a pre-operative biopsy taken from an osteosarcoma in the left proximal fibula of a 10-year-old boy (Table 2, case 9). Histological image shows filigree disorganized woven tumor bone intimately associated with pleomorphic and hyperchromatic tumor cells (magnification: ×200).

Pre-operative biopsies were acquired in ten patients (15.2%) to decide on neoadjuvant chemotherapy or amputation. A biopsy was performed in one patient with a benign tumor with suspected malignancy, in three patients with a benign aggressive tumor, and in six patients with a malignancy, including four patients with osteosarcoma (Table 2). Exemplary radiographs of osteosarcoma – the most prominent form of malignant bone tumors – in a 10-year-old male patient (case 9) are presented in Figure 2, and the corresponding histology is presented in Figure 3. The biopsy rate was 54.5% in malignant tumors, 13.0% in benign aggressive tumors, and 3.1% in benign tumors. The diagnosis rendered on the basis of pre-operative biopsy corresponded to the diagnosis based on post-surgical resection specimens in all ten cases. Benign aggressive and malignant tumors were 11.2-fold more likely to be biopsied than benign tumors (95% CI: 1.1-63.1; *P* = 0.013; Table 3). Males were 11.2-fold more likely to undergo biopsy than females (95% CI: 1.3-94.1; *P* = 0.013; Table 3). Time from onset to presentation, laterality, symptoms and signs, and symptom changes were not significantly associated with subjecting the patient to a biopsy (Table 3).

**Table 2:** Medical profile of patients whose tumor was biopsied

Case	Age (years)/gender/laterality	Histopathological diagnosis	Pre-operative chemo	Biopsy mode	Surgical approach	Recurrence	Peroneal nerve injury, pre-op/iatrogenic/recovery	Impaired wound healing
1	15/M/R	Osteochondroma	-	Incisional	Marginal	-	+/+/+	-
2	16/M/L	Osteoblastoma and ABC	-	Incisional	Type I	-	-/-/NA	-
3	27/M/R	Giant cell tumor	-	Core needle	Type I	-	-/-/NA	-
4	43/F/R	Chondrosarcoma	-	Incisional	Type I	-	-/-/NA	-
5	16/M/L	Osteosarcoma	+	Core needle	Type I	+	+/-/+	+
6	22/M/L	Osteosarcoma	-	Core needle	Type II	-	+/+/-	+
7	18/M/R	Osteosarcoma	+	Core needle	Type II	-	+/+/-	+
8	8/M/R	Ewing’s sarcoma	+	Core needle	Type II	-	+/+/-	+
9	10/M/L	Osteosarcoma	+	Core needle	Type I	+	-/-/NA	-
10	20/M/R	Giant cell tumor and ABC	-	Core needle	Type I	-	-/-/NA	-

ABC: Aneurysmal bone cyst, NA: Not applicable

**Table 3:** Association of factors with pre-operative biopsy

	Biopsy (*n*=10)	No biopsy (*n*=56)	*P*-value*	Relative risk	95% confidence interval	*P*-value**
Continuous variables [median (range)]						
Age (years)	17 (8-43)	19.5 (4-72)	0.320			
Time from onset to presentation (months)	3 (0-12)	3 (0-108)	0.739			
Categorical variables*** [*n* (percentage)]						
Gender						
Male	9 (26.5)	25 (73.5)		11.2	1.3-94.1	**0.013**
Female (reference)	1 (3.1)	31 (96.9)				
Laterality						
Right	6 (22.2)	21 (77.8)		2.5	0.6-9.9	0.295
Left (reference)	4 (10.3)	35 (89.7)				
Symptoms and signs						
Yes	10 (17.9)	46 (82.1)		N/A	N/A	0.338
No (reference)	0 (0.0)	10 (100.0)				
Symptom change						
Yes	2 (15.4)	11 (84.6)		1.0	0.2-5.5	1.000
No (reference)	8 (15.1)	45 (84.9)				
Histopathological diagnosis						
Malignant and benign aggressive	9 (26.5)	25 (73.5)		11.2	1.1-63.1	**0.013**
Benign (reference)	1 (3.1)	31 (96.9)				

* Mann–Whitney U-test.

** Fisher’s exact test (2-sided).

*** Continuity correction applied to relative risk calculation

In the biopsied patient group, four osteosarcoma patients received neoadjuvant chemotherapy before surgery. The peroneal nerve was sacrificed in three patients during surgery. Patients who had undergone biopsy were 12.4 times more likely to receive Type I or Type II *en bloc* resection (*P* = 0.006) and had a 7.6-fold greater chance to have impaired wound healing (*P* = 0.040) (Table 4). Biopsy did not significantly increase the risk of tumor recurrence (*P* = 0.162) and iatrogenic peroneal nerve injury (*P* = 0.095) (Table 4).

**Table 4:** Association between biopsy and intervention, recurrence, and surgical complications

Biopsy	En bloc resection (Type I or Type II)		Recurrence		Iatrogenic peroneal nerve injury		Impaired wound healing	
			
	Yes	No	Yes	No	Yes	No	Yes	No
Yes	9 (90.0)	1 (10.0)	2 (20.0)	8 (80.0)	3 (30.0)	7 (70.0)	3 (30.0)	7 (70.0)
No (reference)	24 (42.1)	33 (57.9)	3 (5.4)	53 (94.6)	5 (8.9)	51 (91.1)	3 (5.4)	53 (94.6)
Relative risk	12.4		4.4		4.4		7.6	
95% Confidence interval	1.5-104.3		0.6-30.7		0.9-22.4		1.3-45.1	
*P*-value (Fisher exact test)	**0.006**		0.162		0.095		**0.040**	

Values represent *n* (percentage). Continuity correction was applied to relative risk calculation.

## 4. Discussion

Incisional and percutaneous biopsies are commonly performed to increase the diagnostic accuracy with respect to suspected malignant musculoskeletal tumors and to subsequently formulate an appropriate intervention. Recent studies have demonstrated that percutaneous core needle biopsies, particularly those performed under image guidance, constitute a valid alternative to incisional biopsy for diagnosing primary bone tumors [9,10]. Percutaneous biopsies have been widely employed for diagnosing suspected primary malignant bone tumors in the extremities, pelvis, and spine. A Mayo Clinic study encompassing 112 cases of malignant proximal fibular tumors revealed that biopsy-based diagnosis diverted patients to radiation therapy and/or chemotherapy instead of surgery or amputation [11]. Nevertheless, biopsy-based diagnostics for proximal peroneal malignant tumors are performed rather scarcely. Unlike the femur or humerus, segmental resection of proximal fibular tumors does not require bone reconstruction. For benign aggressive tumors, Type I *en bloc* resection can be performed without biopsy because it preserves the peroneal nerve and hence limits the impact on knee function. Given the possible complications from biopsy, the proportion of proximal tumor biopsies is generally lower than expected. In our study cohort, biopsy-based diagnosis was performed in 54.5% of patients with malignant tumors and in 15% of patients with proximal fibular tumors. The relatively low biopsy rate in proximal fibular tumor cases may emanate from concerns related to an increased risk of iatrogenic peroneal nerve injury and other complications as well as the possibility of tumor recurrence.

Iatrogenic peripheral nerve injuries have received increased attention due to their (socio)medical ramifications [12]. In the present study, biopsy did not directly or indirectly lead to iatrogenic peroneal nerve injury. In fact, there are no cases of proximal fibular tumors reported in biopsy-related peroneal nerve injury to date. It has been suggested that biopsy of proximal fibula lesions should be performed by an anterolateral approach: through the safe area, the palpable anterior, to the fibular head, and downward laterally, in the compartment of the peroneus longus muscle bounded by the head of the fibula and the deep peroneal nerve [4,13]. The use of this method is safe even in a proximal fibular tumor with cortical or soft expansion because the peroneal nerves are displaced posteriorly and distally, which may enlarge the safe area posterolaterally. Since fine needle biopsy does not pose a significant risk in tumor seeding [14], a fine needle is recommended for biopsies of proximal fibula lesions to avoid contamination and hematoma formation [4]. However, core needle biopsy is more accurate than fine needle biopsy. Thus, as the risk and difficulty of re-biopsy of proximal fibular lesions are dramatically higher than that of the first biopsy, core needle biopsy is preferred by others [15-17].

The present study showed that biopsy was related to impaired wound healing. However, we speculate that the biopsy in itself may not be the cause of impaired wound healing, but that the impaired wound healing is caused by the malignancy. Our previous study found that malignancy was an independent risk factor for impaired wound healing by multivariate analysis [18].

Biopsies of proximal fibular lesions in our patient cohort always yielded accurate diagnosis that was consistent with the diagnosis based on the resected specimens. The reported diagnostic accuracy varies from 77% to 98% for biopsy of bone and soft-tissue lesions [17,19,20]. Although percutaneous core needle biopsy is a safe, minimally invasive, and cost-effective technique for diagnosing bony lesions, incisional biopsy is still considered the gold standard. It has been reported that lesion type, lesion size, and number of specimens were the determining factors for the diagnostic yield of biopsy [17,19]. The biopsy of proximal fibular tumors was likely to produce diagnostic inaccuracies because of its relatively small lesion size and small “safe area” for repeatedly obtaining specimens. Therefore, we performed both percutaneous biopsy and planed incisional biopsy to obtain enough specimens for establishing histological type and grade.

For musculoskeletal tumors, the aim of biopsy is to differentiate between a benign and malignant tumor or to confirm and grade the malignant tumor, which is essential for standard and effective treatment. The present study analyzed the association of potential indicators, including age, sex, laterality, and symptoms with biopsy, and found that the only significant indicator of biopsy was suspicion of malignant and benign aggressive tumors. Although the pre-biopsy diagnosis was made by well-trained senior orthopedic surgeons based on clinical features, imaging findings, laboratory test results, and their experiences, a pre-surgical biopsy was considered only when the diagnosis was equivocal with respect to malignancy.

Biopsy of proximal fibular lesions is a diagnostic tool to better stratify patients for surgical intervention. In the present study, a patient with osteochondroma with the possibility of becoming malignant underwent biopsy and received marginal resection of the tumor instead of segmental resection of the proximal fibula. This patient developed unstable knee joint and other surgery-related complications. According to a study at the Mayo Clinic, 8% of patients had an incisional biopsy only, followed by radiation therapy and/or chemotherapy [11]. Because osteosarcoma is the most common malignant tumor in the proximal fibula [3,11], and the validity of neoadjuvant chemotherapy for high-grade osteosarcoma is commonly accepted, it is essential to perform biopsy to establish histological type and grade when considering osteosarcoma. In fact, only a small number of patients with osteosarcoma in the proximal fibula received neoadjuvant chemotherapy according to previous studies and this study [11,21]. Some researchers stress the importance of an adequate surgical margin during initial surgery (to circumvent palliative chemotherapy) because they believe the biological behavior of osteosarcoma in the proximal fibula may differ from that of conventional osteosarcoma [21]. Given the paucity of literature, there is no adequate evidence that patients may benefit from not undergoing neoadjuvant chemotherapy. Most studies have reached a consensus that for treatment of osteosarcoma, neoadjuvant chemotherapy is still essential for high-grade osteosarcoma [22,23].

The present study was limited by a relatively small case number due to the sporadic occurrence of these tumors. Only 2.5% of primary bone tumors are located in the fibula, whereby tumors in the proximal fibula are even more rare. Our study included 66 patients, which is average-sized cohort for proximal fibular tumors. Of the 66 included patients, only ten patients had received pre-operative biopsy. The conclusions drawn in this study should therefore be contextualized to the very small sample size, especially with respect to the part on biopsies. Larger-cohort follow-up studies should be conducted to validate the results; although, we acknowledge that this may require long inclusion times. In the interim, reliance on other studies, such as those referenced in this paper, to comprise a comprehensive and optimal treatment plan for proximal fibular cancer patients is strongly encouraged in light of the paucity of available data and the risk for peri-operative and post-operative complications. Second, the percutaneous core needle biopsy and incisional biopsy were analyzed together. Although core needle biopsy was performed more commonly in diagnosis of bone lesions, it was clustered together with incisional biopsy due to small simple size.

## 5. Conclusions

The present study evinced that a core needle biopsy of proximal fibular lesions is an accurate diagnostic tool without increasing the risk of iatrogenic peroneal nerve injury and tumor recurrence. Most proximal fibular tumors are benign, while malignant tumors are rare but life-threatening. Patients with the latter tumor types are more likely to be biopsied, especially when male and when osteosarcoma is suspected. With the histological type and grade of malignancy revealed through biopsy, patients can be better stratified for individualized intervention.

### Conflict of interest

MH is chief formulation officer at Nurish.Me and Camelina Sun and has equity in those companies (whose business activities are unrelated to the present work). The authors declare that they have no competing interests.

### Funding information

TS was supported by the Medical Scientific Research Foundation of Hebei Province, China (grant # 20190648) and the Natural Science Foundation of Hebei Province of China (grant # H2019206609).

MH was supported by grants from the Dutch Cancer Foundation (KWF project # 10666), a Zhejiang Provincial Foreign Expert Program Grant, Zhejiang Provincial Key Natural Science Foundation of China (#Z20H160031), and a grant for the establishment of the Jiaxing Key Laboratory for Photonanomedicine and Experimental Therapeutics.
